# The effects of the COVID-19 pandemic on neuropsychiatric symptoms in dementia and carer mental health: an international multicentre study

**DOI:** 10.1038/s41598-022-05687-w

**Published:** 2022-02-14

**Authors:** Grace Wei, Janine Diehl-Schmid, Jordi A. Matias-Guiu, Yolande Pijnenburg, Ramon Landin-Romero, Hans Bogaardt, Olivier Piguet, Fiona Kumfor

**Affiliations:** 1 Brain and Mind Centre, The University of Sydney, Sydney, Australia; 2 School of Psychology, The University of Sydney, Sydney, Australia; 3 Department of Psychiatry and Psychotherapy, School of Medicine, Technical University of Munich, Munich, Germany; 4 Department of Neurology, Institute of Neurosciences, Hospital Clínico San Carlos, San Carlos Health Research Institute (IdISSC), Madrid, Spain; 5 Department of Neurology, Alzheimer Center Amsterdam, Amsterdam Neuroscience, Vrije Universiteit Amsterdam, Amsterdam UMC, Amsterdam, The Netherlands; 6 School of Allied Health and Practice, The University of Adelaide, Adelaide, Australia

**Keywords:** Human behaviour, Disease prevention, Health policy, Public health, Neurological disorders, Infectious diseases, Neurological disorders

## Abstract

As a global health emergency, the rapid spread of the novel coronavirus disease (COVID-19) led to the implementation of widespread restrictions (e.g., quarantine, physical/social distancing measures). However, while these restrictions reduce the viral spread of COVID-19, they may exacerbate behavioural and cognitive symptoms in dementia patients and increase pressure on caregiving. Here, we aimed to assess the impact of COVID-19 and related restrictions on both carers and people living with dementia across the world. We conducted an international survey (Australia, Germany, Spain, and the Netherlands) to assess the impact of COVID-19 on carers and people living with dementia. People with dementia experienced worsened neuropsychiatric symptoms since the outbreak of COVID-19, most commonly, depression, apathy, delusions, anxiety, irritability, and agitation. Regression analyses revealed that limited understanding of the COVID-19 situation and not living with the carer was associated with worsened neuropsychiatric symptoms. Carers also reported a decline in their own mental health, increased stress and reduced social networks as a result of COVID-19 and related restrictions. Regression analyses revealed uncertainty about the future and loneliness were associated with worsened carer mental health. Findings from this study will inform strategies for the development of support services and compassionate protocols that meet the evolving needs of those living with dementia and their carers.

## Introduction

Environmental variables can have a profound impact on people living with dementia and their carers. This has become acutely evident following the identification of the novel severe acute respiratory syndrome coronavirus (SARS-CoV-2) in late 2019. On March 11, 2020, with more than 118,000 cases identified across 114 countries, the World Health Organisation (WHO) declared the coronavirus disease (COVID-19) a pandemic^1^. As a global health emergency, the rapid spread of COVID-19 led to implementation of widespread restrictions (e.g., quarantine, physical/social distancing measures).

People living with dementia are at a higher risk of contracting COVID-19 and are also more likely to experience more serious, and often lethal consequences of the disease^2–5^. In addition to the vulnerability of people with dementia contracting the disease, COVID-19 has led to public health measures such as quarantine, social distancing and visitation bans in long-term care facilities, that have severely curtailed social contact with family members and carers. Similarly, loss of respite and community services, and reduced hospital visits have resulted in the removal of essential sources of support for those living at home^6^.

Difficulties understanding public health information and adhering to safeguard procedures (e.g., maintaining physical distancing, washing hands, wearing masks) have made managing in lockdown or quarantine situations extremely challenging^7^. Notably, behavioural symptoms of dementia such as disinhibition (e.g., approaching or touching strangers), and stereotyped behaviour (e.g., strict adherence to routine), particularly when coupled with a lack of insight, will further exacerbate pressure on caregiving and increase vulnerability to COVID-19^8^. Individuals with language symptoms, such as in progressive non-fluent aphasia (PNFA) and semantic dementia (SD), may also face additional communication challenges due to mask wearing, as well as reduced understanding of semantic concepts such as virus, mask, or soap.

Early studies have shown that the COVID-19 pandemic and related restrictions have negatively impacted both people with dementia and their carers. Since the outbreak of COVID-19, worsening cognitive function, neuropsychiatric symptoms and functional decline in people with dementia has been reported^9–17^. In an Italian cohort, Canevelli et al. found that 54.7% of people with dementia experienced worsened neuropsychiatric symptoms, with worsened agitation, apathy and depression the most commonly observed^11^. Similarly, carers have reported increased levels of burden, anxiety, depression, and distress during the COVID-19 pandemic^9,10,12,13,16,17^. To date however, studies have largely been conducted in single-country cohorts, wherein comparisons across countries are limited and lack standardised assessment. Further, studies have largely only examined more common forms of dementia such as Alzheimer’s disease (AD), with the impact on people living with frontotemporal dementia (FTD) underrepresented. Here, we aimed to assess the impact of COVID-19 and related restrictions on neuropsychiatric symptoms across diverse cohorts of people with dementia as well as the impact on carer mental health, in Australia, Germany, Spain and the Netherlands. In line with previous findings, we hypothesised that difficulties adapting to restrictions and reduced support since the outbreak of COVID-19 would result in worsened psychological outcomes for both people with dementia and their carers.

## Methods

### Participants

Carers of individuals with dementia were invited to complete an online survey hosted on a secure web-based database (Research Electronic Data Capture (REDCap) between April and November 2020. The survey collected information from carers about the person with dementia including demographics, clinical information and neuropsychiatric symptoms, both *before* and *after* the outbreak of COVID-19, using an abbreviated version of the Neuropsychiatric Inventory^18^, as well as information about the carers’ own demographics, their perceptions and the impact of COVID-19 on their social network and mental health (see Supplementary Information). Time of survey completion in relation to COVID-19 was calculated based upon the number of days since 100 cases of COVID-19 was reported in the respondents’ respective country, as a common point across countries^19^.

German, Spanish, and Dutch versions of the survey were translated by native speakers (by J.D-S, R.L-R and H.B, respectively) and back translated into English to ensure consistency across versions prior to data collection. Participants were recruited from the FRONTIER dementia research clinic in Sydney, Australia; the Center for Cognitive Disorders, Technical University Munich and the German and Munich Alzheimers’ Associations in Germany; the Hospital Clínico San Carlos in Madrid, Spain; and the Amsterdam University Medical Center and FTD Lotgenoten (Dutch FTD patient society) in the Netherlands. In addition, the study was shared on social media platforms affiliated with the institutions and organizations listed above to allow for snowball recruitment.

### Statistical analyses

Data were analysed using SPSS (IBM, Version 26.0). Descriptive statistics were used to summarise the data, as mean and standard deviation (SD) or frequency (percentage). Differences between countries on the prevalence of neuropsychiatric symptoms were analysed using Chi-Square tests, with Bonferroni correction for multiple comparisons. Binary logistic regression analyses (enter method) were used to identify demographic, clinical and COVID-19 specific characteristics independently associated with worsened neuropsychiatric symptoms, operationalized as responses of “(b) worsened” respectively since the outbreak of COVID-19, and carer mental health since the outbreak of COVID-19. Regression analyses were conducted for neuropsychiatric symptoms that were reportedly worsened in over 25% of the study cohort, with Bonferroni correction. Data from the Netherlands (Dutch version) were not included in the regression analyses due to small sample size. For all analyses, statistical significance was set at *p* < 0.05.

### Ethics approval

Participation was voluntary and all participants provided informed consent in accordance with the Declaration of Helsinki and its later amendments. This study was approved by the University of Sydney Human Research Ethics Committee (2020/213).

## Results

### Demographic information

Demographic and clinical characteristics of the study cohort are reported in Table 1. 287 carers of people with dementia participated in the study (67 respondents in Australia, 78 in Germany, 119 in Spain and 23 in the Netherlands). Respondents completed the survey after a mean period of 109.26 days since 100 cases of COVID-19 were reported in their respective countries^18^. 128 (44.6%) of the carers were spouses, 131 (45.6%) were children of the individual with dementia, and 28 (9.8%) were classified as other (i.e., sibling, friend, niece, nephew, ex-spouse or unspecified).

**Table 1:** Demographic and clinical characteristics of study cohort.

	All (n = 287)	Australia (n = 67)	Germany (n = 78)	Spain (n = 119)	The Netherlands (n = 23)
Survey completion (Days since 100 cases)	109.26 ± 48.57	52.06 ± 15.38	138.72 ± 49.51	109.40 ± 19.43	175.30 ± 41.79
Age (Carer)	57.21 ± 12.63	60.58 ± 12.07	53.27 ± 11.80	56.79 ± 13.22	62.45 ± 9.44
Sex (Carer), Female (n)	215 (74.9%)	46 (68.7%)	62 (79.5%)	87 (73.1%)	20 (87.0%)
Region (Carer), Urban (n)	192 (66.9%)	38 (56.7%)	46 (59.0%)	108 (90.8%)	N/A
Living with (Carer), No (n)	133 (46.3%)	19 (28.4%)	47 (60.3%)	52 (43.7%)	15 (65.2%)
Caring for children (Carer), No (n)	215 (74.9%)	53 (79.1%)	54 (69.2%)	91 (76.5)	17 (73.9%)
Age (Patient)	73.79 ± 10.37	70.83 ± 8.71	74.15 ± 11.78	76.69 ± 9.71	66.75 ± 8.24
Sex (Patient), female (n)	159 (55.4%)	32 (47.8%)	45 (57.7%)	79 (66.4%)	3 (13.0%)
**Relationship (Carer)**					
Spouse (n)	128 (44.6%)	44 (65.7%)	28 (35.9%)	37 (31.1%)	19 (82.6%)
Child (n)	131 (45.6%)	16 (23.9%)	42 (53.8%)	71 (59.7%)	2 (8.7%)
Other (n)	28 (9.8%)	7 (10.4%)	8 (10.3%)	11 (9.2%)	2 (8.7%)
**Diagnosis (Patient)**					
AD (n)	120 (41.8%)	17 (25.4%)	32 (41.0%)	71 (59.7%)	0 (0.0%)
FTD (n)	125 (43.6%)	37 (55.2%)	30 (38.0%)	36 (30.3%)	22 (95.7%)
Other dementia (n)	42 (14.6%)	13 (19.4%)	16 (20.5%)	12 (10.1%)	1 (4.3%)

Values are mean ± standard deviation, unless otherwise stated. Region refers to the area respondents are living in (urban or regional area). Living with refers to whether respondents are living with the person they care for (yes or no). *AD* Alzheimer’s disease, *FTD* frontotemporal dementia. Study cohort consists of single carer-patient dyads (i.e. one carer providing care for one patient).

Of the people with dementia, 125 (43.6%) had a diagnosis of FTD, 120 (41.8%) had a diagnosis of AD, and 42 (14.6%) were classified as other dementia [i.e., Lewy body dementia (n = 5), vascular dementia (n = 5), posterior cortical atrophy (n = 3), hydrocephalus (n = 2), Parkinson’s disease (n = 1), Korsakoff’s syndrome (n = 1), or unspecified (n = 25)].

### Neuropsychiatric symptoms

**Figure 1:**
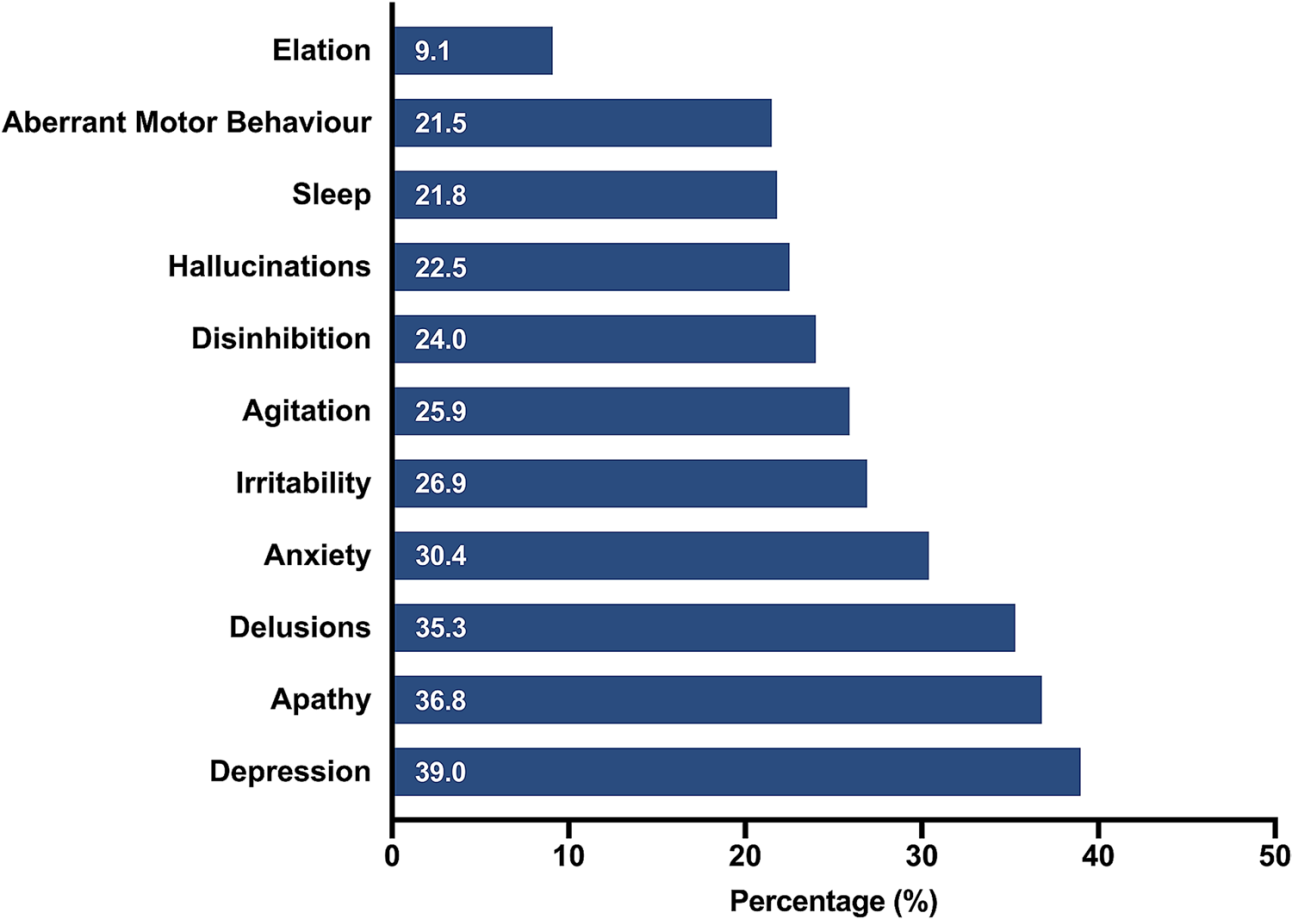
Worsened neuropsychiatric symptoms reported in the overall study cohort following the outbreak of COVID-19.

Prior to the outbreak of COVID-19, the prevalence of neuropsychiatric symptoms did not differ according to country (all *p* values > 0.05, see Supplementary Information). People with dementia showed worsened neuropsychiatric symptoms since the outbreak of COVID-19 (Fig. 1). Depression was the most frequently reported mood disturbance, with 39.0% of people with dementia showing increased depression since the COVID-19 outbreak, whereas increased elation was reported least frequently (9.1%). Apathy was also common, reported in 36.8% of the cohort. In terms of psychotic symptoms, worsened delusions (35.3%) were reported more frequently than hallucinations (22.5%).

### Regression analyses

**Figure 2:**
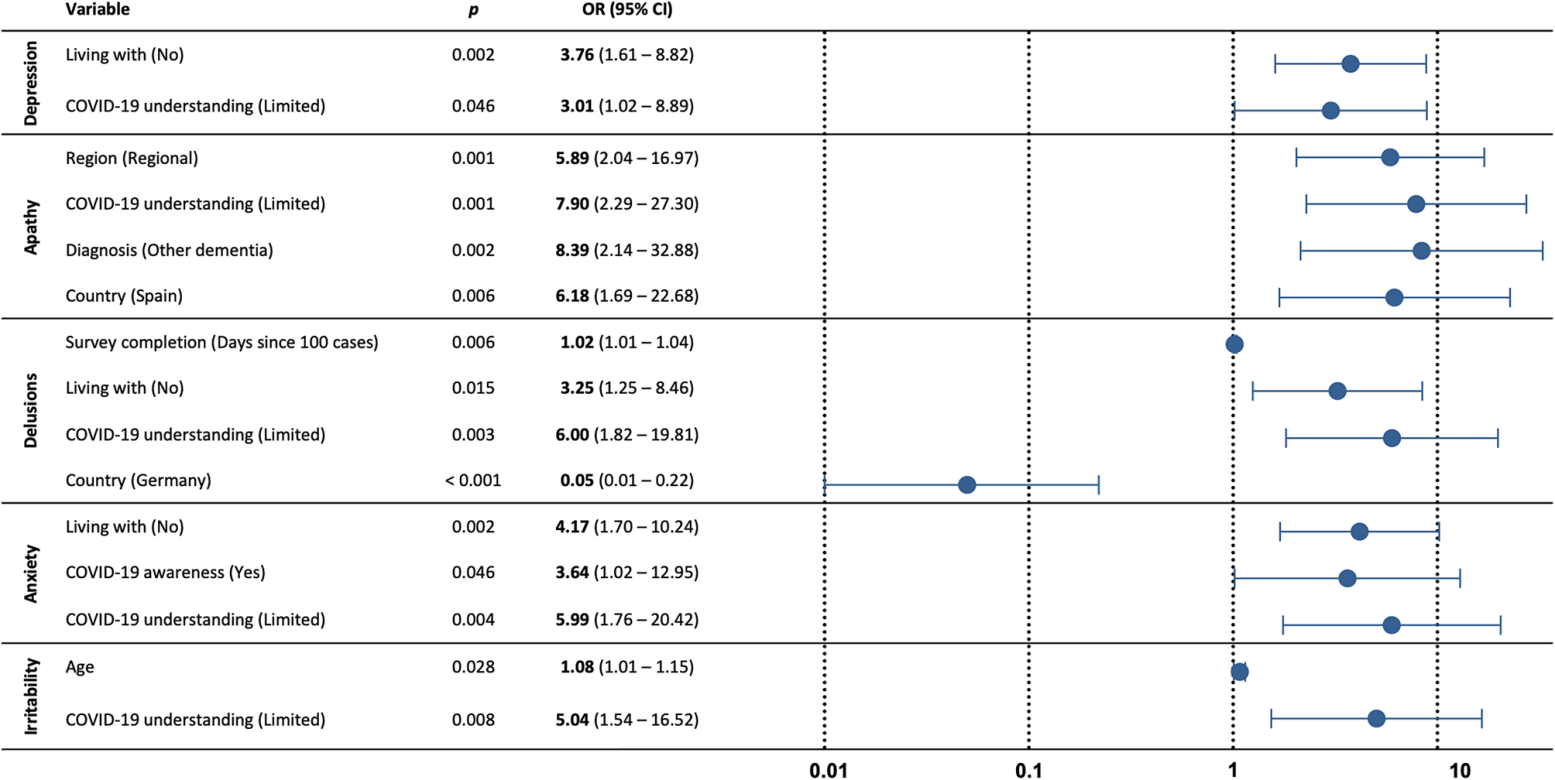
Predictors of worsened neuropsychiatric symptoms: depression (n = 143); apathy (n = 142); delusions (n = 142); anxiety (n = 141); irritability (n = 143). *Data from the Netherlands (Dutch version) were not included in the analyses due to small sample size.

Here, we focused on the most common neuropsychiatric features to have worsened (i.e., in over 25% of the study cohort): depression, apathy, delusions, anxiety, irritability and agitation. We investigated whether demographic, clinical or environmental variables predicted changes in each neuropsychiatric symptom separately. Predictor variables entered into the six regression models were selected a priori, with a Bonferroni-adjusted alpha level of 0.008 (0.05/6). Predictor variables included survey completion (i.e., number of days since 100 cases of COVID-19 was reported in the respective country), the person with dementia’s age, sex, country (Australia, Germany or Spain), region (urban or regional area), diagnosis (AD, FTD or other dementia), whether they were living with the carer, awareness and understanding of COVID-19. Results of the regression analyses are depicted in Fig. 2. Full details of the models are in Table 2.

**Table 2:** Predictors of worsened neuropsychiatric symptoms.

Variable	Depression		Apathy		Delusions		Anxiety		Irritability	
	*p*	OR (95% CI)	*p*	OR (95% CI)	*p*	OR (95% CI)	*p*	OR (95% CI)	*p*	OR (95% CI)
Survey completion (Days since 100 cases)	0.386	0.99 (0.98–1.01)	0.075	1.01 (0.99–1.03)	**0.006**	**1.02 (1.01–1.04)**	0.254	1.01 (0.99–1.02)	0.612	1.00 (0.99–1.02)
Age (Patient)	0.269	1.03 (0.98–1.08)	0.397	1.02 (0.97–1.03)	0.185	1.04 (0.98–1.10)	0.484	1.02 (0.97–1.07)	**0.028**	**1.08 (1.01–1.15)**
**Sex (Patient)**										
Male	–	–	–	**–**	–	–	–	–	–	–
Female	0.436	1.39 (0.61–3.18)	0.294	0.60 (0.23–1.56)	0.111	0.47 (0.18–1.19)	0.984	0.96 (0.38–2.44)	0.067	0.40 (0.15–1.07)
**Country**										
Australia	0.745	0.80 (0.20–3.15)	0.076	5.64 (0.83–38.10)	0.340	2.01 (0.48–8.37)	0.417	0.56 (0.13–2.30)	0.336	2.06 (0.47–8.96)
Germany	0.141	0.42 (0.13–1.33)	–		**0.000**	**0.05 (0.01–0.22)**	0.100	0.36 (0.11–1.22)	0.068	0.25 (0.06–1.11)
Spain	–	–	**0.006**	**6.18 (1.69–22.68)**	–	–	–	–	–	–
**Region**										
Urban	–	–	–	–	–	–	–	–	–	–
Regional	0.097	2.15 (0.87–5.29)	**0.001**	**5.89 (2.04–16.97)**	0.174	2.06 (0.73–5.84)	0.508	1.40 (0.52–3.78)	0.987	0.99 (0.34–2.93)
**Diagnosis**										
AD	0.362	0.57 (0.17–1.92)	–	–	0.215	0.43 (0.11–1.63)	0.503	1.43 (0.50–4.06)	0.639	1.45 (0.31–6.77)
FTD	0.910	0.93 (0.27–3.27)	0.705	0.81 (0.27–2.42)	0.181	0.39 (0.10–1.56)	0.171	0.39 (0.10–1.51)	0.456	1.84 (0.37–9.07)
Other dementia	–	–	**0.002**	**8.39 (2.14–32.88)**	–	–	–	–	–	–
**Living with**										
Yes	–	–	–	–	–	–	–	–	–	–
No	**0.002**	**3.76 (1.61–8.82)**	0.477	1.40 (0.56–3.51)	**0.015**	**3.25 (1.25–8.46)**	**0.002**	**4.17 (1.70–10.24)**	0.399	1.52 (0.58–4.02)
**COVID-19 awareness**										
Yes	0.299	0.54 (0.16–1.74)	0.748	0.82 (0.25–2.69)	0.481	0.64 (0.18–2.24)	**0.046**	**3.64 (1.02–12.95)**	0.140	0.39 (0.11–1.36)
No	–	**–**	–	–	–	–	–	–	–	–
**COVID-19 understanding**										
None/limited	**0.046**	**3.01 (1.02–8.89)**	**0.001**	**7.90 (2.29–27.30)**	**0.003**	**6.00 (1.82–19.81)**	**0.004**	**5.99 (1.76–16.52)**	**0.008**	**5.04 (1.54–16.52)**
Moderate/good	–	–	–	–	–	–	–	–	–	–

Significant values are in bold.

Responses of “unsure” were not included in the regression analyses.

For depression, 21.8% of variance was explained by the model (*p* = 0.008). The only significant predictors were not living with the carer (OR 3.76, *p* = 0.002) and a limited understanding of COVID-19 (OR 3.01, *p* = 0.046).

For apathy, 32.7% of variance was explained by the model (*p* < 0.001). Living in a regional area (OR 5.89, *p* = 0.001) and a limited understanding of COVID-19 (OR 7.90, *p* = 0.001) were independently associated with an increased risk of worsened apathy. Those with other forms of dementia were more likely to have worsened apathy (OR 8.39, *p* = 0.002) than those with Alzheimer’s disease (AD). People with dementia in Spain were more likely to have worsened apathy (OR 6.18, *p* = 0.006) compared to those in Germany.

For delusions, 34.1% of variance was explained by the model (*p* < 0.001). Longer duration since 100 cases of COVID-19 were reported in the respective country (OR 1.02, *p* = 0.006), not living with the carer (OR 3.25, *p* = 0.015) and a limited understanding of COVID-19 (OR 6.00, *p* = 0.003) were independently associated with an increased risk of worsened delusions. People with dementia in Germany were less likely to have worsened delusions (OR 0.05, *p* < 0.001) compared to those in Spain.

For anxiety, 25.3% of variance was explained by the model (*p* = 0.003). Not living with the carer (OR 4.17, *p* = 0.002), awareness of COVID-19 (OR 3.64, *p* = 0.046), but limited understanding of COVID-19 (OR 5.99, *p* = 0.004) were independently associated with an increased risk of worsened anxiety.

For irritability, 25.0% of variance was explained by the model (*p* = 0.006). Increasing age (OR 1.08, *p* = 0.028) and a limited understanding of COVID-19 (OR 5.04, *p* = 0.008) were independently associated with an increased risk of worsened irritability.

The regression model for agitation was not significant (*p* = 0.024).

Carers reported worsened neuropsychiatric symptoms in people with dementia since the outbreak of the COVID-19 pandemic. Worsened depression, apathy, delusions, anxiety, irritability, and agitation were the most reported. Worsening of these symptoms was associated with limited awareness and understanding of COVID-19, not living with the carer, duration of the pandemic, age, dementia diagnosis, country and living in a regional area.

### Carer mental health

**Figure 3:**
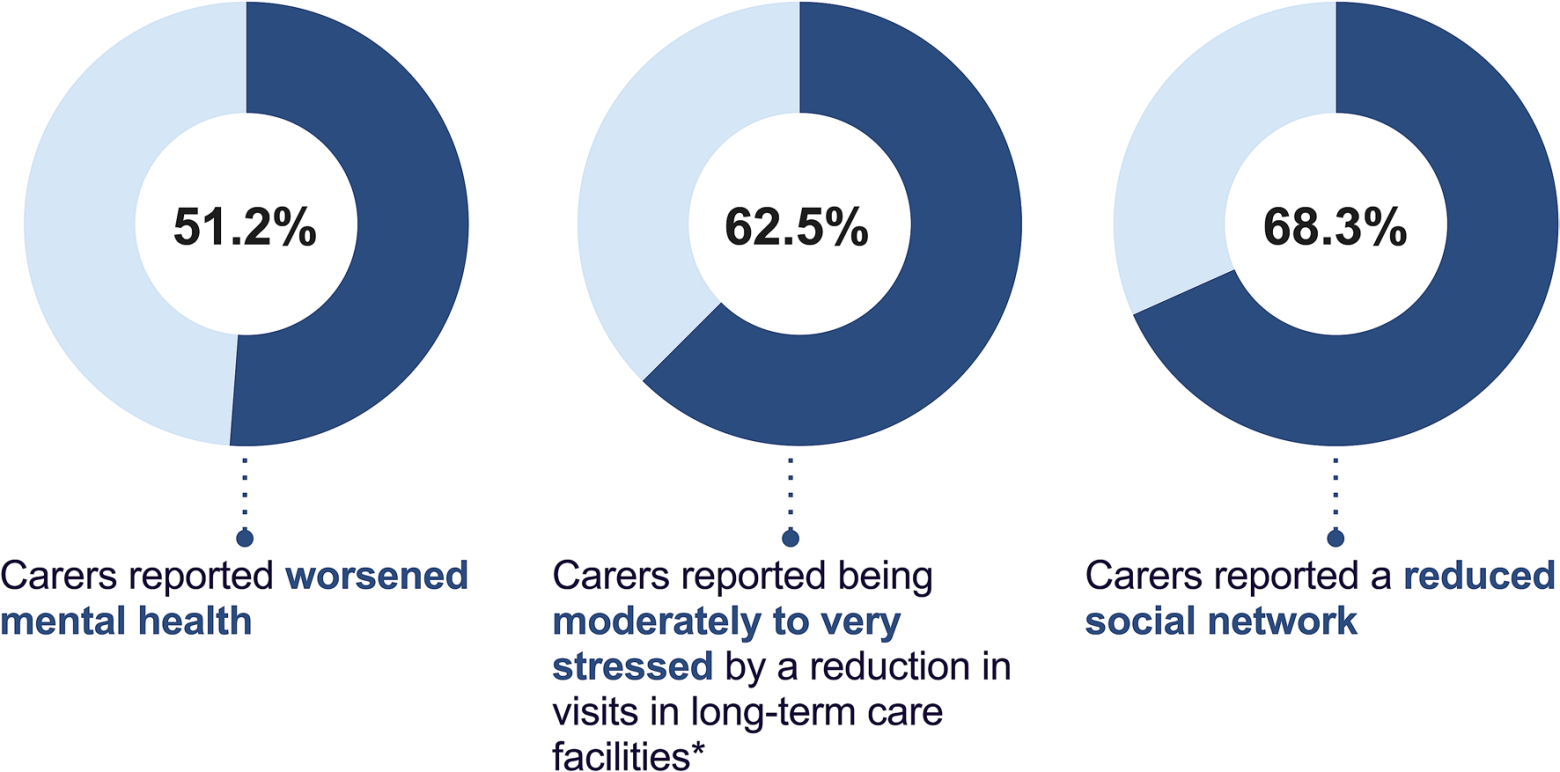
Worsened carer mental health, increased stress and reduced social network reported in the study cohort. *Carers caring for an individual with dementia living in a long-term care facility (n = 96).

Carers reported psychological changes and a reduced social network since the outbreak of COVID-19 (Fig. 3). Over half of the carers (51.2%) reported they had worsened mental health and 68.3% reported a reduced social network since the outbreak of COVID-19. Among the carers caring for a person with dementia living in a long-term care facility, 62.5% reported being moderately to very stressed by a reduction in visits.

#### Regression analyses

Here, we focused on changes in carer mental health. Predictor variables entered into the regression analyses were survey completion (i.e., number of days since 100 cases of COVID-19 was reported in the respective country), the carer’s age, sex, country (Australia, Germany or Spain), region (urban or regional area), whether they were caring for children, how much information they had consumed about COVID-19, their confidence in their knowledge of COVID-19, their concern about finances and concerns about the future since the outbreak of COVID-19, their social network, and loneliness, as well as their relationship and whether they were living with the person they care for, and diagnosis of the person with dementia (AD, FTD or other dementia).

**Figure 4:**
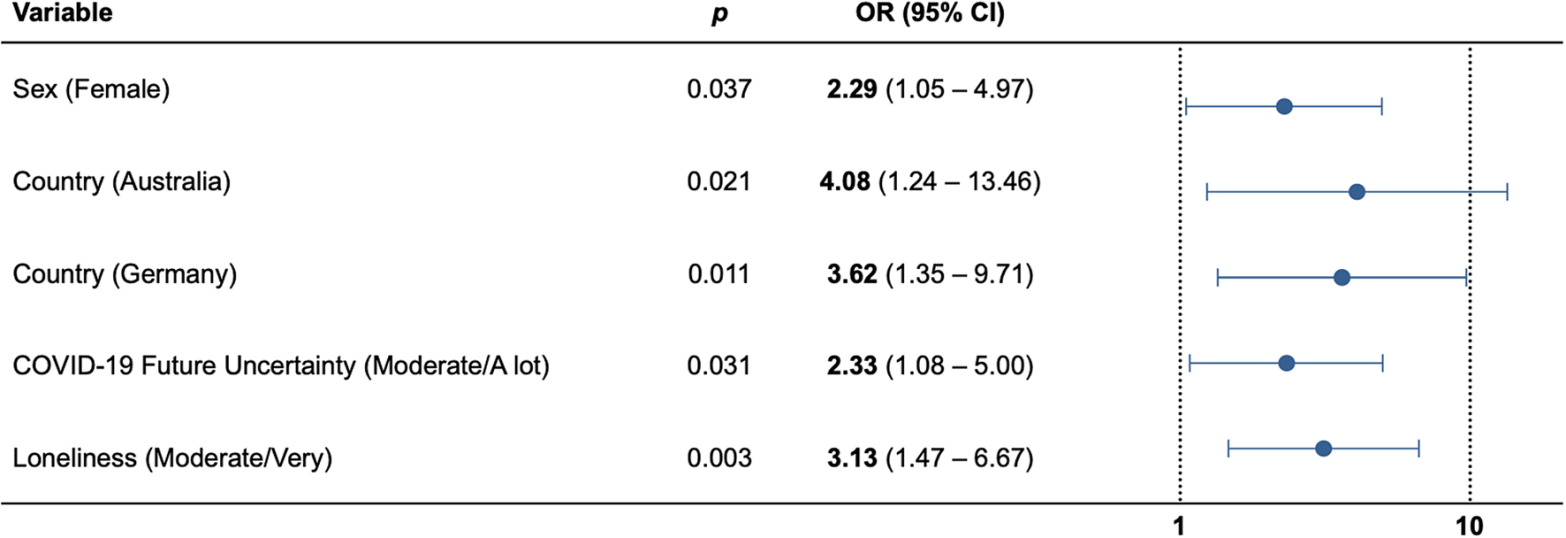
Predictors of worsened carer mental health (n = 193). *Data from the Netherlands (Dutch version) were not included in the analysis due to the small sample size.

The logistic regression model was able to explain 27.7% of variance in worsened carer mental health since the outbreak of COVID-19 (*p* < 0.001) (Fig. 4 and Table 3). Female carers were more likely to report worsened mental health (OR 2.29, *p* = 0.037). Carers in Australia and Germany were more likely to report worsened mental health (OR 4.08, *p* = 0.021; OR 3.62, *p* = 0.011, respectively) compared to those in Spain. Being moderately or a lot concerned about the future since the outbreak of COVID-19 was associated with an increased likelihood of worsened carer mental health (OR 2.33, *p* = 0.031). Carers who reported being moderately to very lonely had an increased risk of worsened mental health (OR 3.13, *p* = 0.003).

**Table 3:** Predictors of worsened carer mental health.

Variable	*p*	OR (95% CI)
Survey completion (Days since 100 cases)	0.765	1.00 (0.99–1.01)
Age	0.477	0.99 (0.95–1.03)
**Sex**		
Male	–	–
Female	**0.037**	**2.29 (1.05–4.97)**
**Country**		
Australia	**0.021**	**4.08 (1.24–13.46)**
Germany	**0.011**	**3.62 (1.35–9.71)**
Spain	–	–
**Region**		
Urban	–	–
Regional	0.504	1.33 (0.58–3.04)
**Childcare**		
Yes	0.799	0.89 (0.37–2.13)
No	–	–
**Diagnosis**		
AD	0.932	0.96 (0.34–2.73)
FTD	0.401	0.63 (0.22–1.85)
Other dementia	–	–
**Relationship**		
Spouse	0.790	0.84 (0.23–3.07)
Child	0.548	0.70 (0.21–2.27)
Other	–	–
**Living with**		
Yes	0.890	0.94 (0.41–2.19)
No	–	–
**COVID-19 information**		
None/little	–	–
Moderate/a lot	0.282	1.57 (0.69–2.56)
**COVID-19 confidence**		
None/little	–	–
Moderate/a lot	0.234	0.49 (0.15–1.58)
**COVID-19 financial**		
None/little	–	–
Moderate/a lot	0.653	0.83 (0.36–1.91)
**COVID-19 future**		
None/little	–	–
Moderate/a lot	**0.031**	**2.33 (1.08–5.00)**
**Social network (reduced)**		
Yes	0.493	1.31 (0.61–2.80)
No	–	–
**Loneliness**		
None/little	–	–
Moderate/very	**0.003**	**3.13 (1.47–6.67)**

Significant values are in bold.

Carers reported experiencing worsened mental health, reduced social networks and increased stress since the outbreak of the COVID-19 pandemic. Worsened carer mental health was associated with the carer’s sex, country, uncertainty about the future and loneliness.

## Discussion

This study aimed to investigate the impact of the COVID-19 pandemic and related restrictions on people living with dementia and their carers, in Australia, Germany, Spain and the Netherlands. We found worsened neuropsychiatric symptoms in people with dementia, most commonly depression, apathy, delusions, anxiety, irritability, and agitation. Carers reported worsened mental health, increased levels of stress and decreased social networks. In the following sections, we consider which mechanisms contribute to these detrimental psychological effects and potential ways to minimise harm in these vulnerable populations.

The number of people living with dementia has more than doubled in the last 20 years worldwide, posing an increasing burden on family carers, and health-care systems^20^. Here, we examine the nature of two global health challenges in tandem, dementia, and the COVID-19 pandemic. While recent epidemiological studies suggest that the prevalence of dementia is relatively similar (1.82% in Australia; 1.91% in Germany; 1.83% in Spain and 1.49% in the Netherlands)^21,22^, cultural factors, and differences in health policy and resources across countries play an important role in dementia support and care. Notably, in the context of this study, Spain has been one of the hardest hit by the COVID-19 pandemic^19^. Existing regional disparities in service accessibility and funding of aged care services, as well as heavy reliance on informal family carers, with up to 80% of all people with dementia living in the community, have been further exacerbated by the pandemic^23,24^. Universally, however, people living with dementia have been disproportionately affected by the COVID-19 pandemic, as evidenced by higher rates of mortality and negative social consequences of public health measures which have resulted in visitation bans and loss of dementia support services worldwide^2–6,25^.

The restrictions implemented as a result of the COVID-19 pandemic pose challenges to people living with dementia. In our cohort, patients showed worsened neuropsychiatric symptoms, with worsened depression the most frequently reported symptom (39.0%) and increased elation relatively uncommon (9.1%). This profile of mood disturbances parallels findings in an Italian cohort^11^. In line with previous reports^26^, worsened apathy (36.8%), anxiety (30.4%) and agitation (25.9%) were also common. Unlike previous studies however^10,14,26^, we found a relatively high incidence of delusions and hallucinations (35.3% and 22.5% respectively). The mechanisms underlying the increased prevalence of these psychotic symptoms warrant further investigation in future studies. Taken together, our findings contribute to growing evidence of more severe neuropsychiatric disturbances in people living with dementia as a direct result of the COVID-19 pandemic.

The regression analyses revealed that limited understanding of COVID-19 in people with dementia was found to be a significant predictor for the five most frequent neuropsychiatric symptoms examined. Awareness in combination with limited understanding of COVID-19, was associated with worsened anxiety. Impaired comprehension of the public health information regarding COVID-19 has previously been reported^7^, and as shown here, appears to have a direct impact on neuropsychiatric disturbances. Notably, these findings suggest that interventions to improve health literacy and understanding through campaigns and education to support both patients’ and carers are critical in the context of the rapidly changing COVID-19 environment.

People with dementia not living with the carer (e.g., in long-term care facilities) were more likely to experience worsened depression, delusions, and anxiety. Of relevance here, these symptoms may be exacerbated by a reduction in meaningful contact with the carer due to COVID-19 related restrictions such as quarantine or lockdown, and visitation bans in long-term care facilities^27,28^. Other demographic and clinical variables were also involved in these models but less consistently. For example, increased age was associated with worsened irritability. Several studies have shown that irritability becomes more frequent and severe with disease progression^29–31^. Those with other forms of dementia were more likely to experience worsened apathy than those with Alzheimer’s disease (AD). However, given the variability in these syndromes classified here as *other*, including mostly unspecified dementia, meaningful interpretation of this finding is limited. Despite no longstanding differences in apathy prior to the outbreak of COVID-19, regional differences emerged. People with dementia in Spain were more likely to experience worsened apathy than those in Germany. There was also less incidence of worsened delusions in Germany compared to Spain. Of note, the COVID-19 pandemic has hit the hardest in Spain relative to other countries included in this study^19^, with lockdown measures modified several times in response to multiple waves^23^. In Spain, disrupted routines, and reduced access to support emerged as a result of widespread shortage of personal protective equipment which led to the suspension of home care services, closure of day centers and visitation bans in long-term care facilities^24^. Although, across countries, a longer duration since 100 cases of COVID-19 was reported was associated with an increased risk of worsened delusions. Overall, our results indicate that greater support for people with dementia is urgently needed. Person-centered care is critical during the COVID-19 pandemic and should seek to improve understanding of COVID-19 and related public health measures, maintain cognitive and physical stimulation and meaningful social connections^32^.

We also found that carers reported worsened mental health and a reduction in their social networks since the outbreak of COVID-19. Of relevance here, Altieri et al., reported increased depressive symptomology, but not anxiety, in carers during lockdown^9^. The nature of worsened mental health and the psychological effects of the COVID-19 pandemic should be further examined in future studies using cross-culturally validated screening tools. Those caring for someone living in a long term or residential care facility also reported moderate to high levels of stress as a result of a reduction in the number of visits. Our findings suggest that visitation bans in these facilities may have inadvertently led to significant psychological harm. Recent calls to change visitation policies have been made by experts in the field^33–35^. Our data provide empirical support to reconsider visitation bans and find alternate means to protect vulnerable individuals from virus transmission while also considering the psychological impact of isolation. Public health measures and restrictions to mitigate the spread of COVID-19 have resulted in prolonged periods of separation from loved ones, as well as reduced access to regular systems of support including health and respite services, and social support (e.g., from extended family and friends)^6,36^. The additional challenges compound the burden and stress already experienced by many carers^37,38^.

In line with previous research, we found that female carers were more likely to report worsened mental health than males^39,40^. Interestingly, carers in Australia and Germany were more likely to report worsened mental health since the outbreak of COVID-19, compared to carers in Spain. Culturally relevant norms with regards to familial obligations and responsibilities play an important role in caregiving. Greater intergenerational solidarity (e.g., co-resident living arrangements) and familial support networks in Spain may mitigate the risk of worsened carer mental health^41^. We also found that greater uncertainty of the future was associated with worsened carer mental health. Of relevance here, rapidly changing information and public health measures in relation to COVID-19 can heighten feelings of uncertainty about the future. Carers of people with dementia living in long-term care facilities may also experience increased uncertainty about the future as a result of visitation bans and disproportionately high rates of infection and death in long-term care residents worldwide^25,42^. Worsened carer mental health since the outbreak of COVID-19 was also associated with greater loneliness. Increased feelings of isolation stem from the introduction of mandatory isolation (i.e. quarantine/lockdown) and reductions in visits to long term care facilities^43^. Social isolation and loneliness have been shown to contribute to negative psychological health outcomes including increased carer burden and depression^39,44^. Determining particularly vulnerable carers is crucial so that targeted interventions and support services may be developed to ameliorate the impact of the COVID-19 pandemic.

Findings from this study should be considered with some limitations in mind. Given the nature of survey-based research, some data were missing. Respondents with missing data in one or more of the predictor variables were not included in the regression analyses. Given the organisational constraints of recruiting a multi-lingual cohort in a pandemic setting where face-to-face testing was not possible, patients were not cognitively assessed in the present study. Formal estimates of disease duration or measures of disease severity were not captured, which may have additional influences on the development of neuropsychiatric symptoms in people with dementia. Factors such as not living with the carer and a limited understanding of the COVID-19 situation offer insight into how dementia severity and disease duration may contribute to worsened outcomes. Future studies should consider use of formalised cognitive assessment and measures of dementia severity and disease duration where feasible.

Given the pandemic setting, information about people with dementia were collected from family carers. We also assessed carers’ perceptions and the impact of the COVID-19 pandemic on their own mental health and social network. The relationship between patient and carer outcomes is likely to be bidirectional^45–47^, and further empirical studies are needed to investigate this complex association. While carers’ responses are subjective, our findings are highly consistent with the emerging evidence of higher rates of neuropsychiatric symptoms and negative psychological effects on carers during the pandemic^9–16^. Understanding of COVID-19 situation is also likely to be variable and evolving within the general population. In this study, we did not specifically assess carers’ objective knowledge of COVID-19, which may also influence their perceptions of patients’ understanding of COVID-19. Nevertheless, this large multi-centre international study still arguably provides the most comprehensive investigation of the impact of the COVID-19 pandemic on both people with dementia and their carers, to date.

It is worth noting that the severity (e.g. multiple waves and peaks in case numbers) and extent of the societal response to the COVID-19 pandemic varies across countries and will differ across jurisdictions even within the same country. As such, we have taken into account the number of days since 100 cases of COVID-19 was reported in each country in our analyses, as a common point across countries during the pandemic^19^. While these findings capture a snapshot of the impact, the long-term effects of the COVID-19 pandemic on people with dementia and their carers remain unknown and will be an important avenue for future research.

In summary, we have shown that COVID-19 and related restrictions to mitigate its viral spread, have resulted in worsened neuropsychiatric symptoms in people with dementia, as well as worsened mental health and increased stress in carers worldwide. This population is particularly vulnerable to the negative social consequences of this pandemic, as it is often the first to be subject to strict restrictions and the last to come out of prolonged periods of separation from their loved ones. It is evident that a more considered approach is needed, balancing the risk of COVID-19 infection and ramifications of lockdown measures and social isolation. This study has important clinical implications, highlighting the urgent need for interventions aimed at targeting health literacy and management of psychological and behavioural symptoms in patients, and improving socialisation and psychological support for carers. Findings from this study will inform approaches to sustain care and health services, and the development of compassionate protocols that meet the evolving and complex needs of people living with dementia and their carers.

## Supplementary Information

Supplementary Information.

## Data Availability

The datasets generated and/or analysed during the present study are available from the corresponding author upon reasonable request.

## References

[1] World Health Organisation. *Coronavirus Disease 2019 (COVID-19) Situation Report.* 2020(51).

[2] Bianchetti A (2020). Clinical presentation of COVID19 in dementia patients. J. Nutr. Health Aging.

[3] Atkins JL (2020). Preexisting comorbidities predicting COVID-19 and mortality in the UK biobank community cohort. J. Gerontol. A Biol. Sci. Med. Sci..

[4] Williamson EJ (2020). Factors associated with COVID-19-related death using OpenSAFELY. Nature.

[5] Kuo CL (2020). APOE e4 genotype predicts severe COVID-19 in the UK biobank community cohort. J. Gerontol. A Biol. Sci. Med. Sci..

[6] Giebel C (2020). Impact of COVID-19 related social support service closures on people with dementia and unpaid carers: A qualitative study. Aging Ment. Health.

[7] Wang H (2020). Dementia care during COVID-19. Lancet.

[8] Suzuki M (2020). The behavioral pattern of patients with frontotemporal dementia during the COVID-19 pandemic. Int. Psychogeriatr..

[9] Altieri M, Santangelo G (2021). The psychological impact of COVID-19 pandemic and lockdown on caregivers of people with dementia. Am. J. Geriatr. Psychiatry.

[10] Cagnin A (2020). Behavioral and psychological effects of coronavirus disease-19 quarantine in patients with dementia. Front. Psychiatry.

[11] Canevelli M (2020). Facing dementia during the COVID-19 outbreak. J. Am. Geriatr. Soc..

[12] Carpinelli Mazzi M (2020). Time of isolation, education and gender influence the psychological outcome during COVID-19 lockdown in caregivers of patients with dementia. Eur. Geriatr. Med..

[13] Boutoleau-Bretonniere C (2020). The effects of confinement on neuropsychiatric symptoms in Alzheimer's disease during the COVID-19 crisis. J. Alzheimers Dis..

[14] Lara B (2020). Neuropsychiatric symptoms and quality of life in Spanish patients with Alzheimer's disease during the COVID-19 lockdown. Eur. J. Neurol..

[15] Manini A (2021). The impact of lockdown during SARS-CoV-2 outbreak on behavioral and psychological symptoms of dementia. Neurol. Sci..

[16] Tsapanou A (2020). The impact of COVID-19 pandemic on people with mild cognitive impairment/dementia and on their caregivers. Int. J. Geriatr. Psychiatry.

[17] Rainero I (2020). The impact of COVID-19 quarantine on patients with dementia and family caregivers: A nation-wide survey. Front. Aging Neurosci..

[18] Cummings JL (1997). The Neuropsychiatric Inventory: Assessing psychopathology in dementia patients. Neurology.

[19] Dong E, Du H, Gardner L (2020). An interactive web-based dashboard to track COVID-19 in real time. Lancet Infect. Dis..

[20] G. B. D Dementia Collaborators (2019). Global, regional, and national burden of Alzheimer’s disease and other dementias, 1990–2016: A systematic analysis for the Global Burden of Disease Study 2016. Lancet Neurol..

[21] Alzheimer Europe. *Dementia in Europe Yearbook 2019: Estimating the Prevalence of Dementia in Europe*. 108 (Alzheimer Europe, 2019).

[22] Australian Institute of Health and Welfare, *Dementia*. (2020).

[23] Burns A (2021). COVID-19 and dementia: Experience from six European countries. Int. J. Geriatr. Psychiatry.

[24] Suarez-Gonzalez, A., Matias-Guiu, J. A. & Comas-Herrera, A. *Impact and Mortality of the First Wave of COVID-19 on People Living with Dementia in Spain*. LTCcovid, International Long-Term Care Policy Network, CPEC-LSE (2020).

[25] Matias-Guiu JA, Pytel V, Matias-Guiu J (2020). Death rate due to COVID-19 in Alzheimer's disease and frontotemporal dementia. J. Alzheimers Dis..

[26] Simonetti A (2020). Neuropsychiatric symptoms in elderly with dementia during COVID-19 pandemic: Definition, treatment, and future directions. Front. Psychiatry.

[27] Numbers K, Brodaty H (2021). The effects of the COVID-19 pandemic on people with dementia. Nat. Rev. Neurol..

[28] Manca R, De Marco M, Venneri A (2020). The impact of COVID-19 infection and enforced prolonged social isolation on neuropsychiatric symptoms in older adults with and without dementia: A review. Front. Psychiatry.

[29] Srikanth S, Nagaraja AV, Ratnavalli E (2005). Neuropsychiatric symptoms in dementia-frequency, relationship to dementia severity and comparison in Alzheimer's disease, vascular dementia and frontotemporal dementia. J. Neurol. Sci..

[30] Tanaka H (2015). Relationship between dementia severity and behavioural and psychological symptoms in early-onset Alzheimer's disease. Psychogeriatrics.

[31] Kazui H (2016). Differences of behavioral and psychological symptoms of dementia in disease severity in four major dementias. PLoS ONE.

[32] O'Shea E (2020). Remembering people with dementia during the COVID-19 crisis. HRB Open Res..

[33] Dementia Australia. *One Day the Support was Gone: The Mental Health Impact of COVID-19 on People Living with Dementia, Their Families and Carers*. (Dementia Australia, 2020).

[34] Verbeek H (2020). Allowing visitors back in the nursing home during the COVID-19 crisis: A Dutch National Study into first experiences and impact on well-being. J. Am. Med. Dir. Assoc..

[35] Low, L.-F. *et al.**Safe Visiting at Care Homes During COVID-19: A Review of International Guidelines and Emerging Practices During the COVID-19 Pandemic.* LTCcovid, International Long-Term Care Policy Network, CPEC-LSE (2021).

[36] Brown EE (2020). Anticipating and mitigating the impact of the COVID-19 pandemic on Alzheimer's disease and related dementias. Am. J. Geriatr. Psychiatry.

[37] Baumgarten M (1992). The psychological and physical health of family members caring for an elderly person with dementia. J. Clin. Epidemiol..

[38] Campbell P (2008). Determinants of burden in those who care for someone with dementia. Int. J. Geriatr. Psychiatry.

[39] Bookwala J, Schulz R (2000). A comparison of primary stressors, secondary stressors, and depressive symptoms between elderly caregiving husbands and wives: The Caregiver Health Effects Study. Psychol. Aging.

[40] Pinquart M, Sorensen S (2003). Associations of stressors and uplifts of caregiving with caregiver burden and depressive mood: A meta-analysis. J. Gerontol. B Psychol. Sci. Soc. Sci..

[41] Sole-Auro A, Crimmins EM (2014). Who cares? A comparison of informal and formal care provision in Spain, England and the USA. Ageing Soc..

[42] Cordasco F (2020). The silent deaths of the elderly in long-term care facilities during the Covid-19 pandemic: The role of forensic pathology. Med. Leg J..

[43] Smith BJ, Lim MH (2020). How the COVID-19 pandemic is focusing attention on loneliness and social isolation. Public Health Res. Pract.

[44] Coen RF (1997). Behaviour disturbance and other predictors of carer burden in Alzheimer's disease. Int. J. Geriatr. Psychiatry.

[45] Kwok YT (2011). Assessment of behavioral and psychological symptoms of dementia by family caregivers. Arch. Gerontol. Geriatr..

[46] Tan EY (2021). Interaction of caregiver-expressed emotions and neuropsychiatric symptoms in persons with dementia: A longitudinal cohort study. BMJ Open.

[47] Alexopoulos P (2021). COVID-19 crisis effects on caregiver distress in neurocognitive disorder. J. Alzheimers Dis..

