# Supplementary material for: The effects of the COVID-19 pandemic on neuropsychiatric symptoms in dementia and carer mental health: an international multicentre study
Source: Sci Rep. 2022 Feb 14;12:2418. doi: 10.1038/s41598-022-05687-w (PMC8844310; doi:10.1038/s41598-022-05687-w)
Supplement: Supplementary file 1 — Supplementary Information. [file 41598_2022_5687_MOESM1_ESM.pdf]

The effects of the COVID-19 pandemic on neuropsychiatric symptoms in dementia and carer mental health: an international multicentre study

Grace Wei[1,2], Janine Diehl-Schmid[3], Jordi A. Matias-Guiu[4], Yolande Pijnenburg[5], Ramon Landin-Romero[1,2], Hans Bogaardt[6], Olivier Piguet[1,2], Fiona Kumfor[1,2]

[1] The University of Sydney, Brain and Mind Centre, Sydney, Australia
[2] The University of Sydney, School of Psychology, Sydney, Australia
[3] Technical University of Munich, School of Medicine, Department of Psychiatry and Psychotherapy, Munich, Germany
[4] Hospital Clínico San Carlos, Department of Neurology, Institute of Neurosciences, San Carlos Health Research Institute (IdISSC), Madrid, Spain
[5] Alzheimer Center Amsterdam, Department of Neurology, Amsterdam Neuroscience, Vrije Universiteit Amsterdam, Amsterdam UMC, Amsterdam, The Netherlands
[6] The University of Adelaide, School of Allied Health and Practice, Adelaide, Australia

SUPPLEMENTARY MATERIALS

COVID-19 Survey (*English version*)

Demographics

Please answer the following questions about yourself.

1. Age:
2. Gender:
    a. Female
    b. Male
    c. Do not wish to disclose
3. Country:
    a. Australia
    b. Other *(please specify)*
4. State:
    a. Australian Capital Territory
    b. New South Wales
    c. Northern Territory
    d. Queensland
    e. South Australia
    f. Tasmania
    g. Victoria
    h. Western Australia
    i. Other *(please specify)*
5. Region:
    a. Urban area
    b. Regional area
6. Country born:
7. First language:
8. Employment status *(select all that apply)*:
    - Full-time
    - Part-time
    - Casual
    - Self-employed
    - Independent contractor
    - Student
    - Retired
    - Seeking work
    - Pension
    - Disability benefits
    - Unemployed due to COVID-19
    - Other (*please specify*)
9. Are you caring for children:
    a. Yes > If 'Yes', *please specify child's age*
    b. No

Demographics about the person you care for

Please answer the following questions about the person you care for.

10. Diagnosis:
    a. Alzheimer's disease
    b. Frontotemporal dementia > If 'Frontotemporal dementia', *please specify variant (if known)*
    c. Other dementia > If 'Other dementia', *please specify*
11. Age:
12. Gender:
    a. Female
    b. Male
    c. Do not wish to disclose
13. Country:

    a. Australia
    b. Other *(please specify)*

14. State:
    a. Australian Capital Territory
    b. New South Wales
    c. Northern Territory
    d. Queensland
    e. South Australia
    f. Tasmania
    g. Victoria
    h. Western Australia
    i. Other *(please specify)*
15. Region:
    a. Urban area
    b. Regional area
16. Country born:
17. First language:
18. Languages other than English known:
    a. Yes > If Yes, *please specify*
    b. No
19. What is your relationship to the person you care for:
    a. Spouse
    b. Child
    c. Sibling
    d. Friend
    e. Other > If 'Other', *please specify your relationship to the person you care for*
20. Are you currently living with the person you care for:
    a. Yes
    b. No > If 'No', *please specify* hours spent caring per week
21. Prior to the outbreak of COVID-19, how often did you visit the person you care for:
    a. Not at all
    b. Daily
    c. Every other day
    d. Weekly
    e. Other (*please specify*)
22. In the last week, how often did you visit the person you care for:
    a. Not at all
    b. Daily
    c. Every other day
    d. Weekly
    e. Other (*please specify*)
23. If there has been a reduction in the number of visits, how stressful has this been for you:
    a. Not stressful at all
    b. A little stressful
    c. Moderately stressful
    d. Very stressful
    e. N/A – No reduction in number of visits
24. If there has been a reduction in the number of visits, how stressful do you think this has been for the person you care for:
    a. Not stressful at all
    b. A little stressful
    c. Moderately stressful
    d. Very stressful
    e. N/A – No reduction in number of visits

Perceptions and impacts of COVID-19

25. In the past week, how much information have you heard, read or seen about COVID-19:
    a. None at all (0 hours per week)

    b. A little (Less than 10 hours per week)
    c. A moderate amount (10-20 hours per week)
    d. A lot (More than 20 hours per week)
26. How confident are you about your knowledge about COVID-19:
    a. Not at all
    b. A little
    c. A moderate amount
    d. A lot
27. Since the outbreak of COVID-19, how worried have you been about your financial situation:
    a. Not at all
    b. A little
    c. A moderate amount
    d. A lot
28. Since the outbreak of COVID-19, how uncertain have you been feeling about the future:
    a. Not at all
    b. A little
    c. A moderate amount
    d. A lot
29. Is the person you care for aware of the current situation regarding COVID-19:
    a. Yes
    b. No
    c. Unsure
30. Does the person you care for understand the current situation regarding COVID-19:
    a. They have a limited understanding
    b. They have a moderate understanding
    c. They have a good understanding
    d. Unsure

Social network and COVID-19

31. Prior to the outbreak of COVID-19, how many people did you see or talk to on a regular basis (at least once every 2 weeks), including family, friends, colleagues, neighbours, etc:
    a. 0
    b. 1
    c. 2
    d. 3
    e. 4
    f. 5
    g. 6
    h. 7 or more
32. Since the outbreak of COVID-19, how many people do you see or talk to on a regular basis (at least once every 2 weeks), including family, friends, colleagues, neighbours, etc:
    a. 0
    b. 1
    c. 2
    d. 3
    e. 4
    f. 5
    g. 6
    h. 7 or more
33. Since the outbreak of COVID-19, how have you connected with your social network (select all that apply):
    - Telephone
    - Videotelephony (e.g. Video calls, Facetime, Skype, Zoom, etc.)
    - Messaging (e.g. Text messaging, Facebook Messenger, WhatsApp, etc.)
    - Email
    - Other (*please specify*)
34. Since the outbreak of COVID-19, how much do you feel you can rely on your social network:
    a. Not at all

    b. A little
    c. A moderate amount
    d. A lot

Current situation

35. Are you currently in self-isolation?
    a. Yes – voluntary self-isolation (i.e. if you are following general government advice)
    b. Yes – mandatory self-isolation (i.e. if you are required to self-isolate at the request of Border Force authorities, state police or the health department).
    c. No
36. Please select the answer which best describes *your* current situation:
    a. I have been diagnosed with COVID-19 in the past and am now recovered
    b. I currently have COVID-19 (confirmed with diagnostic tests)
    c. I suspect I have COVID-19 (not confirmed with diagnostic tests)
    d. I do not have COVID-19 and have not experienced it
    e. Unsure
    f. Other *(please specify)*
37. Please select the answer which best describes the current situation of *the person you're caring for*:
    a. They have been diagnosed with COVID-19 in the past and have now recovered
    b. They currently have COVID-19 (confirmed with diagnostic tests)
    c. They suspect they have COVID-19 (not confirmed with diagnostic tests)
    d. They do not have COVID-19 and have not experienced it
    e. Unsure
    f. Other *(please specify)*

Mental health

38. Prior to the outbreak of COVID-19, my mental health was:
    a. Poor
    b. Average
    c. Excellent
39. Since the outbreak of COVID-19, my mental health has been:
    a. A lot worse
    b. A little worse
    c. Stayed the same
    d. A little better
    e. A lot better
40. Are you currently receiving any treatment for depression or anxiety (e.g. medications, counselling, therapy)
    a. Yes
    b. No
    c. Prefer not to say
41. Prior to the outbreak of COVID-19, how lonely did you feel:
    a. Not lonely at all
    b. Average
    c. Very lonely
42. Since the outbreak of COVID-19, how lonely have you felt?
    a. Not lonely at all
    b. A little lonely
    c. Moderately lonely
    d. Very lonely

Behaviours and management

43. Prior to the outbreak of COVID-19, did the person you care for have beliefs that you know are not true? (Delusions)
    a. Yes
    b. No
    c. N/A

    Since the outbreak of COVID-19, how has this changed? (Delusions)
    a. No change
    b. Worsened

    c. Improved

44. Prior to the outbreak of COVID-19, did the person you care for seem to see, hear or experience things that were not present? (Hallucinations)
    a. Yes
    b. No
    c. N/A

    Since the outbreak of COVID-19, how has this changed? (Hallucinations)
    a. No change
    b. Worsened
    c. Improved
45. Prior to the outbreak of COVID-19, did the person you care for have periods when he/she refused to cooperate or wouldn’t let people help him/her? (Agitation)
    a. Yes
    b. No
    c. N/A

    Since the outbreak of COVID-19, how has this changed? (Agitation)
    a. No change
    b. Worsened
    c. Improved
46. Prior to the outbreak of COVID-19, did the person you care for seem sad or depressed? (Depression)
    a. Yes
    b. No
    c. N/A

    Since the outbreak of COVID-19, how has this changed? (Depression)
    a. No change
    b. Worsened
    c. Improved
47. Prior to the outbreak of COVID-19, was the person you care for very nervous, worried or frightened for no apparent reason? (Anxiety)
    a. Yes
    b. No
    c. N/A

    Since the outbreak of COVID-19, how has this changed? (Anxiety)
    a. No change
    b. Worsened
    c. Improved
48. Prior to the outbreak of COVID-19, did the person you care for seem too cheerful or too happy for no apparent reason? (Elation)
    a. Yes
    b. No
    c. N/A

    Since the outbreak of COVID-19, how has this changed? (Elation)
    a. No change
    b. Worsened
    c. Improved
49. Prior to the outbreak of COVID-19, did the person you care for lack interest or motivation for engaging in activities or conversation? (Apathy)
    a. Yes
    b. No
    c. N/A

    Since the outbreak of COVID-19, how has this changed? (Apathy)
    a. No change
    b. Worsened
    c. Improved
50. Prior to the outbreak of COVID-19, did the person you care for seem to act impulsively without thinking? (Disinhibition)

    a. Yes
    b. No
    c. N/A

    Since the outbreak of COVID-19, how has this changed? (Disinhibition)

    a. No change
    b. Worsened
    c. Improved

51. Prior to the outbreak of COVID-19, did the person you care for get irritated and easily disturbed?
    a. Yes
    b. No
    c. N/A

    Since the outbreak of COVID-19, how has this changed? (Irritability)

    a. No change
    b. Worsened
    c. Improved

52. Prior to the outbreak of COVID-19 did the person you care for pace, do things over and over such as opening closets or drawers, or repeatedly pick at things or wind strings or threads? (Aberrant motor behaviour)
    a. Yes
    b. No
    c. N/A

    Since the outbreak of COVID-19, how has this changed? (Aberrant motor behaviour)

    a. No change
    b. Worsened
    c. Improved

53. Did the person you care for have difficulty sleeping? (Sleep)
    a. Yes
    b. No
    c. N/A

    Since the outbreak of COVID-19, how has this changed? (Sleep)

    a. No change
    b. Worsened
    c. Improved

54. Since the outbreak of COVID-19, have your responses to these behaviours changed? Please specify.

The prevalence of neuropsychiatric symptoms prior to the outbreak of COVID-19 did not differ according to country (Delusions, $\chi^2(3) = 6.625$, $p = 0.085$; hallucinations, $\chi^2(3) = 1.263$, $p = 0.738$; agitation, $\chi^2(3) = 8.556$, $p = 0.036$; depression, $\chi^2(3) = 2.293$, $p = 0.514$; anxiety, $\chi^2(3) = 3.304$, $p = 0.347$; elation, $\chi^2(3) = 3.396$, $p = 0.941$; apathy, $\chi^2(3) = 3.843$, $p = 0.279$; disinhibition, $\chi^2(3) = 7.845$, $p = 0.049$; irritability, $\chi^2(3) = 9.324$, $p = 0.025$; aberrant motor behaviour, $\chi^2(3) = 2.815$, $p = 0.421$; sleep disturbance, $\chi^2(3) = 4.719$, $p = 0.194$).

Note. Bonferroni correction for multiple comparisons, $p < 0.005$ (0.05/11).
